# Cholangio-Conundrum: A Case Series of Painless Jaundice

**DOI:** 10.1089/crpc.2015.29002.apj

**Published:** 2015-11-01

**Authors:** Adam P. Johnson, Deviney A. Rattigan, Michael J. Pucci, Jeffrey P. Baliff, Jordan M. Winter, Charles J. Yeo, Harish Lavu

**Affiliations:** ^1^ Department of Surgery, The Jefferson Pancreas, Biliary and Related Cancer Center, Thomas Jefferson University, Philadelphia, Pennsylvania.; ^2^ Department of Pathology, Anatomy, & Cell Biology, Thomas Jefferson University, Philadelphia, Pennsylvania.

**Keywords:** cholangiocarcinoma, Mirizzi syndrome, painless jaundice, pancreatic cancer

## Abstract

**Background:** Correct preoperative diagnosis of hepatobiliary disease can be challenging—even with current advances in radiographical imaging, laboratory testing, and endoscopic evaluation.

**Case presentation 1:** A 65-year-old female with painless jaundice and weight loss was found to have cholelithiasis complicated by the Mirizzi syndrome.

**Case presentation 2:** A 71-year-old female with new-onset painless jaundice and impacted stone in the gallbladder neck was found to have a cholangiocarcinoma.

**Case presentation 3:** A 70-year-old male with progressive painless jaundice and weight loss was found to have a pancreatic adenocarcinoma.

**Conclusion:** Proper diagnosis and management of patients with painless jaundice can be difficult in the preoperative setting and may require surgical exploration to obtain a definitive diagnosis.

## Introduction

Insidious obstruction of the extrahepatic bile duct is a common cause of painless jaundice in adult patients. The differential diagnosis of posthepatic biliary obstruction is broad. Malignant causes include periampullary cancers of the pancreas or duodenum, primary cholangiocarcinoma, ampullary carcinoma, gallbladder carcinoma, lymphoma, or extrinsic compression from another gastrointestinal malignancy. Benign causes of painless jaundice often mimick carcinomas and include bile duct strictures secondary to pancreatic pseudocysts or chronic pancreatitis, sclerosing cholangitis, external compression by gallstones in the neck of the gallbladder (Mirizzi syndrome), or intraductal parasites—such as liver flukes or ascariasis. Advances in laboratory, imaging, and endoscopic techniques have improved preoperative diagnostic capabilities.^1^ Despite this, obtaining a definitive diagnosis of certain hepatobiliary disease processes remains challenging. At times, additional highly invasive diagnostic maneuvers, such as diagnostic laparoscopy or even open exploration, are required. Herein, we report three interesting cases with similar presentations and diagnostic evaluations, but vastly different underlying pathologies and management strategies.

## Case 1

**FIG. 1.**
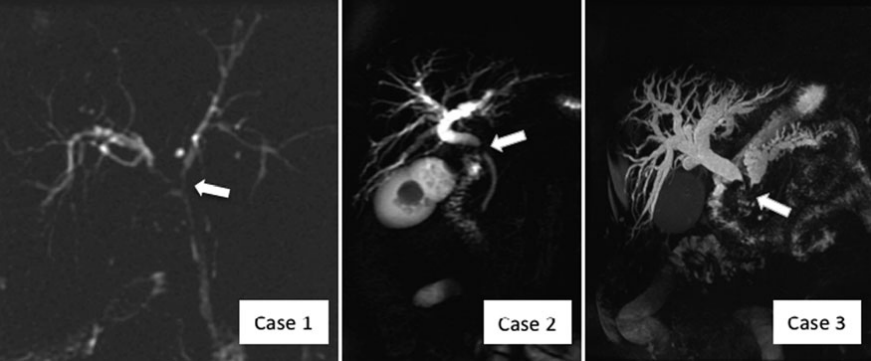
Comparison of magnetic resonance cholangiopancreatography images. Case 1, after placement of 7F stents, showing narrowing of hepatic duct confluence (arrow). Case 2, showing common hepatic duct narrowing at the level of the impacted calculus (arrow). Case 3, showing extrahepatic and intrahepatic bile duct dilatation and significant pancreatic duct dilatation with defect in distal biliary tree (arrow).

A 65-year-old female presented with fatigue, 20 lb weight loss, and intermittent painless jaundice. Initial laboratory investigations revealed an elevated bilirubin of 9.8 mg/dL (normal range 0.3–1.7), alkaline phosphatase of 977 U/L (normal 44–127), and carbohydrate antigen (CA) 19-9 of 267 U/mL (normal 0–37). Abdominal ultrasound revealed cholelithiasis with possible central biliary tree dilation. Endoscopic retrograde cholangiography (ERC) with sphincterotomy showed a malignant-appearing stricture in the common hepatic duct but yielded equivocal brushings and biopsies (Table 1). A laparoscopic cholecystectomy had been attempted at an outside institution but was aborted secondary to extensive inflammation in the porta hepatis, preventing visualization of the gallbladder. After referral to our institution, the patient underwent a repeat ERC and magnetic resonance cholangiopancreatography (MRCP) (Fig. 1) with placement of bilateral 7F biliary stents. Again, duct brushings revealed only benign cells with fibrosis and inflammation. The differential diagnosis included primary gallbladder adenocarcinoma, hilar cholangiocarcinoma, and cholecystitis with extrinsic compression of the biliary tree (Mirizzi syndrome), and the decision was made to proceed with open exploration.

**Table 1.** **Comparison of the Laboratory Results, Imaging, and Final Diagnosis for Cases 1, 2, and 3**

Patient	Case 1: 65-year-old female	Case 2: 71-year-old female	Case 3: 70-year-old male
Bilirubin	Elevated	Initially elevated, but normal after stenting	Initially elevated, but normal after stenting
Alkaline phosphatase	Elevated	Initially elevated, but normal after stenting	Initially elevated, but normal after stenting
CA 19-9	Elevated	Initially elevated, but normal after stenting	Elevated, even after stenting
Ultrasound	Cholelithiasis with possible central biliary tree dilation	Dilation in the intrahepatic and extrahepatic ducts, stone impacted in gallbladder neck	None performed
MRCP	Filling defect at the confluence of the hepatic ducts and central dilation	Common hepatic duct narrowing at the level of the stone	Significant intrahepatic and extrahepatic biliary duct dilatation and dilation of the pancreatic duct. Ill-defined hypoechoic 2.5 × 2.0 cm mass in the head of the pancreas
ERC	Malignant-appearing stricture in the common hepatic duct with dilation of the intrahepatic ductal system	Placement of a 12F biliary endoprosthesis across the biliary stricture	Cutoff in the lower third of the common bile duct, sphincterotomy performed and stent placed
	Biopsy and brushings were inconclusive		Brushings concerning for malignancy (adenocarcinoma)
Final diagnosis	Mirizzi syndrome	Primary cholangiocarcinoma of the common hepatic duct with concurrent impacted gallstone	Pancreatic adenocarcinoma

CA, carbohydrate antigen; ERC, endoscopic retrograde cholangiography; MRCP, magnetic resonance cholangiopancreatography.

**FIG. 2.**
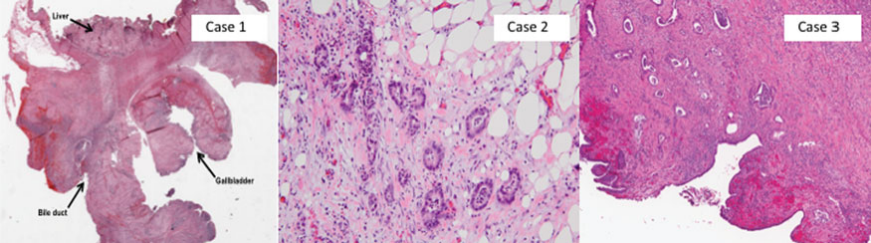
Comparison of pathology. Case 1 on low-powered view (10×, hematoxylin–eosin) showing part of the gallbladder wall with abundant surrounding inflammation and fibrosis extending into the hepatic bed and adjacent bile duct. Case 2 on high-powered view, showing adenocarcinoma (200×, hematoxylin–eosin). Case 3 on medium-powered view (40×, hematoxylin–eosin), showing pancreatic ductal adenocarcinoma involving the bile duct.

During open surgical exploration, the gallbladder was markedly inflamed with dense adhesions to the duodenum, without signs of disease dissemination. On dissection of the gallbladder, an obvious fistula was identified between the infundibulum of the gallbladder and the biliary bifurcation. Intraoperative frozen section analysis was negative for malignancy. After resection of the damaged extrahepatic bile ducts, separate right and left hepatic ducts remained. Reconstruction was performed with bilateral hepaticojejunostomies to a Roux-en-Y segment of the jejunum. Pathological analysis (Fig. 2) of the surgical resection specimen revealed xanthogranulomatous cholecystitis with extensive fibrosis and inflammation (without dysplasia or malignancy), consistent with the Mirizzi syndrome. The patient recovered well and returned to her practice as a clinical psychologist.

## Case 2

A 71-year-old previously healthy female presented with 2 weeks of painless jaundice. Ultrasound evaluation showed dilation of the intrahepatic and extrahepatic bile ducts as well as an impacted calculus in the gallbladder neck. Computed tomography (CT) and MRCP (Fig. 1) confirmed the dilation and revealed the common bile duct narrowing at the level of the impacted stone. Before referral to our institution, endoscopic retrograde cholangiopancreatography (ERCP) was performed with successful placement of a biliary endoprosthesis and resolution of her jaundice. In our clinic, the patient was without complaints and laboratory evaluation revealed a bilirubin of 1.7 mg/dL, an alkaline phosphatase of 226 U/L, and aspartate transaminase and alanine transaminase of 49 and 41 U/L, respectively, and both CA 19-9 and carcinoembryonic antigen (CEA) were within normal limits (Table 1). The differential diagnosis for this patient was identical to that noted in Case 1. The patient was scheduled for an open exploration—cholecystectomy, extrahepatic biliary resection, and Roux-en-Y hepaticojejunostomy.

On operative exploration, a gallstone was found impacted in the neck of the gallbladder. However, a dense mass was also found at the junction of the gallbladder and the bile duct, encasing the right hepatic artery and portal vein—intraoperative frozen section revealed adenocarcinoma. Given the presence of vascular encasement, the most effective palliative measure to drain the biliary tree was determined to be resection of the damaged and partially obstructed bile ducts and performance of a palliative hepaticojejunostomy bypass. Pathological analysis of the resected specimen revealed a moderately differentiated adenocarcinoma, involving the cystic and common hepatic ducts, perineural and angiolymphatic invasion, and metastatic carcinoma in one station 12 lymph node (Fig. 2). After recovery from surgery, the patient was treated with palliative chemoradiotherapy and she survived for an additional 12 months postoperatively.

## Case 3

A 70-year-old male with a history of smoking and long-standing diabetes presented with a 60 lb weight loss, fatigue, constipation, and painless jaundice. Initial MRCP showed significant intrahepatic and extrahepatic biliary ductal dilation. In addition, it also showed a dilated pancreatic duct and ill-defined hypoechoic mass within the head of the pancreas (Fig. 1). The patient underwent biliary stenting at the time of ERCP and a biopsy was performed through endoscopic ultrasound, which was concerning for malignancy. The patient was referred to our institution for further management. On arrival at our institution, the serum bilirubin and alkaline phosphatase had normalized to 1.1 mg/dL and 80 U/L, respectively. His CEA was within normal limits, but the serum CA 19-9 remained elevated at 184 from an initial value of 328 U/mL before his biliary stenting (Table 1). The differential diagnosis for this man was broad, similar to the previous two cases, but due to the distal bile duct obstruction and mass within the head of the pancreas, a primary pancreatic malignancy was strongly suspected.

The patient underwent operative exploration and was found not to have any evidence of disseminated disease. A firm mass was noted involving the head and uncinate process of the pancreas. He underwent a cholecystectomy and pylorus-preserving pancreaticoduodenectomy with standard reconstruction with an invagination pancreaticojejunostomy, hepaticojejunostomy, and end-to-side duodenojejunostomy. Pathological analysis revealed a successful R0 resection of a T3N1M0 moderately differentiated invasive pancreatic ductal adenocarcinoma, invading into the peripancreatic soft tissue, ampulla, duodenal wall, and bile duct (Fig. 2). The patient recovered well after the surgery and was discharged home on postoperative day 5. He is currently 6 weeks from his resection and has fully recovered.

## Discussion

Painless obstructive jaundice has a wide differential that may require open exploration to establish a definitive diagnosis. These three cases illustrate the importance of preoperative counseling and exhaustive evaluation by an interdisciplinary group of endoscopists, radiologists, and surgeons. The etiology of jaundice, a clinical presentation of hyperbilirubinemia, can be categorized as prehepatic, hepatic, or posthepatic. Prehepatic causes of jaundice include spherocytosis, Gilbert's syndrome, sickle cell disease, or thalassemia major. Intrahepatic causes include hepatitis—viral, alcoholic, or drug induced—alcoholic cirrhosis, or primary biliary cirrhosis. Posthepatic jaundice is caused by an interruption of biliary drainage by either intrinsic or extrinsic obstruction.

The accurate diagnosis of the etiology of obstructive jaundice first requires a thorough history and physical examination, focusing on timing and duration of gastrointestinal symptomatology, including pain, nausea, vomiting, and change in bowel habits. Jaundice is often first recognized by friends or family members, rather than the patients themselves. Laboratory studies, including cell counts, blood chemistries, liver function tests, and pancreatic enzymes, are important to target the level of the pathology. If malignancy is suspected, tumor markers—such as alpha-feto protein, CA 19-9, and CEA—are useful in diagnosis, although it must be recognized that they have low sensitivity and specificity.^1^ They are effective, however, in monitoring for recurrence after initial treatment with resection, chemotherapy, or radiation. Following relief of obstruction, CA 19-9 levels will typically normalize. If they remain elevated, this may indicate an underlying malignant pathology.

Cross-sectional imaging techniques, such as CT and MRCP, are essential to elucidating the cause of posthepatic jaundice. MRCP is particularly useful in delineating biliary anatomy. Endoscopy has played a growing role in diagnosis—through ERC, cholangioscopy, and biopsy—and treatment—through stone extraction, sphincterotomy, and stent placement. With regard to diagnosis, MRCP and ERC have comparable sensitivity, specificity, and accuracy for distinguishing malignant from benign bile duct strictures. Nearly a quarter of biliary strictures are the result of a benign etiology, emphasizing the importance of stricture characterization and tissue biopsy. A lengthy stricture with irregular borders or a “shelf sign” is more suggestive of malignancy, whereas a short stricture with regular borders and a smooth taper indicates a benign stricture. A stricture associated with Mirizzi syndrome is usually long and regular, whereas strictures from autoimmune processes such as primary sclerosing cholangitis are often multifocal.

Biliary obstruction caused by compression of the biliary tree due to impacted gallstones in the neck of the gallbladder is a syndrome first described by Pablo Luis Mirizzi in 1948. Mirizzi syndrome is a rare complication, affecting 0.5–2.5% of patients with gallstone disease. Mirizzi syndrome can lead to the development of a cholecystobiliary fistula. Type 1 Mirizzi syndrome describes external compression without development of a fistula, whereas types II–IV describe progressive levels of severity of these fistulae.^1^ ERCP is the diagnostic modality of choice but has limited accuracy ranging widely from 55% to 90% in the published literature.^1^ Elevated levels of CA 19-9 are not common given the underlying biliary obstruction, further confounding the diagnostic dilemma. Of note, on stenting the patient in Case 1, tumor markers returned to within the normal range, whereas in Case 3, they remained elevated. The preoperative diagnosis of Mirizzi syndrome is important when it is possible to aid in surgical planning. Mirizzi syndrome is often differentiated from malignant causes of obstructive jaundice by symptomatology related to the underlying chronic cholecystitis, emphasizing the importance of a thorough history. However, even in retrospect, the patient in Case 1 was unable to describe any of these symptoms.

Correct diagnosis and effective surgical management of Mirizzi syndrome are especially pertinent, considering the strong association with gallbladder carcinoma. Several retrospective and prospective studies have shown an increased incidence of gallbladder carcinoma in Mirizzi patients (5.3–11%) compared to patients with uncomplicated gallstone disease (1%). Some of these malignancies are only identified at the time of final pathological analysis. Although this association has yet to be completely characterized, it is critical to keep in mind when suspecting a possible Mirizzi syndrome case.^2^

Gallbladder cancers and cholangiocarcinoma are relatively rare, accounting for only 0.6% (9810) of new cancer diagnoses in 2012.^3^ Aggressive radical resection has long been the mainstay of therapy; however, only 30% of these cases will qualify for surgical intervention at the time of diagnosis. In addition, given the lack of effective systemic therapy, many patients have a poor prognosis. Identifying patients early who are candidates for margin negative resection is important for long-term survival. Even with surgical resection, 5-year survival for extrahepatic cholangiocarcinoma is only 27–37%. Liver transplantation is a controversial and rarely used therapy for cholangiocarcinoma.

Pancreatic cancer is slightly more common, 2.7% (44,000) of new cancer diagnoses in 2012, but more importantly, it is the fourth leading cause of death from cancer.^3^ As is the case for distal cholangiocarcinoma, curative therapy for pancreatic cancer is only achieved through radical resection through a pancreaticoduodenectomy. However, only 20–30% of patients qualify for resection at the time of diagnosis because of the prevalence of locally advanced and distant disease spread in the majority of patients. Even with successful resection and adjuvant therapy, survival rates remain low at 15–25% at 5 years.

As in the three cases described here, open surgical exploration for patients with obstructive jaundice may be required to obtain a definitive diagnosis. Laparoscopic approaches in this situation have limited usefulness and have been associated with increased morbidity and mortality. Conversion to an open procedure is often required to safely identify the portal structures. A prospective study of 13,023 cholecystectomy patients at the Swiss Association for Laparoscopic and Thoracoscopic Surgery revealed a conversion rate of 74% for Mirizzi syndrome patients.^4^

## Conclusions

Management of Mirizzi syndrome requires a multidisciplinary approach of palliative endoscopic stenting and definitive surgical therapy through open cholecystectomy, removal of stones, and, in the presence of fistulas, biliary enteric anastomosis. Management of cholangiocarcinoma and pancreatic adenocarcinoma necessitates assessment of resectability and risk stratification for surgical management. However, even with the advancements in diagnostic imaging, proper diagnosis and management of these patients may often require open exploration and astute intraoperative decision-making.

## Abbreviations Used

CA: carbohydrate antigen
CEA: carcinoembryonic antigen
CT: computed tomography
ERC: endoscopic retrograde cholangiography
ERCP: endoscopic retrograde cholangiopancreatography
MRCP: magnetic resonance cholangiopancreatography
